# A randomized trial of a lifestyle intervention in obese endometrial cancer survivors: quality of life outcomes and mediators of behavior change

**DOI:** 10.1186/1477-7525-7-17

**Published:** 2009-02-25

**Authors:** Vivian E von Gruenigen, Heidi E Gibbons, Mary Beth Kavanagh, Jeffrey W Janata, Edith Lerner, Kerry S Courneya

**Affiliations:** 1 University Hospitals Case Medical Center, Cleveland, OH, USA; 2 Department of Reproductive Biology, Case Western Reserve University, Cleveland, OH, USA; 3 Department of Nutrition, Case Western Reserve University, Cleveland, OH, USA; 4 Department of Psychiatry, Case Western Reserve University, Cleveland, OH, USA; 5 Department of Physical Education and Recreation, University of Alberta, Edmonton, AB, Canada

## Abstract

**Background:**

To examine the effects of a 6 month lifestyle intervention on quality of life, depression, self-efficacy and eating behavior changes in overweight and obese endometrial cancer survivors.

**Methods:**

Early stage endometrial cancer survivors were randomized to intervention (n = 23) or usual care (n = 22) groups. Chi-square, Student's t-test and repeated measures analysis of variance were used in intent-to-treat analyses. Outcomes were also examined according to weight loss.

**Results:**

Morbidly obese patients had significantly lower self-efficacy, specifically when feeling physical discomfort. There was a significant improvement for self-efficacy related to social pressure (p = .03) and restraint (p = .02) in the LI group. There was a significant difference for emotional well-being quality of life (p = .02), self-efficacy related to negative emotions (p < .01), food availability (p = .03), and physical discomfort (p = .01) in women who lost weight as compared to women who gained weight. Improvement in restraint was also reported in women who lost weight (p < .01).

**Conclusion:**

This pilot lifestyle intervention had no effect on quality of life or depression but did improve self-efficacy and some eating behaviors.

**Trial Registration:**

; NCT00420979

## Background

Endometrial cancer is the most common gynecologic cancer in the United States and obesity is the most significant risk factor for development of the disease [1]. A recent prospective study reported that 68% of women with early endometrial cancer were obese which is markedly increased compared to older reports [2-5]. Adding to this escalation is the increased rate of obesity in the female population (18.1%) [6]. When assessing obesity associated cancers, it appears that endometrial cancer patients are the most morbid as most have stage I disease yet are at significant risk for premature death [7]. While the impact of weight on cancer recurrence does not appear to be a factor in endometrial cancer, obese endometrial cancer survivors have a higher mortality from causes not related to cancer [8]. Endometrial cancer survivors have numerous co-morbidities related to their obesity which include hypertension (HTN), diabetes mellitus (DM), cardiovascular disease (CVD), osteoarthritis (OA) and pulmonary disease [5]. Improving medical co-morbidities through weight management in survivors may lead to improved overall quality of life and survival.

Differences in quality of life (QOL) between obese and non-obese endometrial cancer survivors are related to physical health. In a prospective examination of QOL, general health status, and obesity in women with early stage endometrial cancer, [9] women with a body mass index (BMI) less than 30 (non-obese) had increased physical QOL scores. There was also an inverse relationship between the global physical health composite score and BMI; with higher BMI associated with a declining physical QOL score. A recent cross-sectional survey of Canadian endometrial cancer survivors revealed that those patients with morbid obesity had a QOL score three times lower than women with a normal BMI [10]. This suggests that if weight is decreased, survivors QOL may be improved. What is unknown is the effect a survivorship lifestyle intervention trial may have on QOL, psychological health and eating behavior.

We have previously reported the effects of a six-month lifestyle intervention on weight loss, exercise behavior, and nutrient intake changes in overweight and obese endometrial cancer survivors [11]. We found that a lifestyle intervention program in obese endometrial cancer survivors is feasible and efficacious – resulting in sustained behavior change and weight loss over a one year period. The present query was conducted in order to examine the intervention's effect on QOL outcomes, depression, self-efficacy and eating behavior, possible mediators of behavior change. We hypothesized that a lifestyle intervention program would improve these outcomes in obese endometrial cancer survivors. We also conducted an exploratory analysis comparing women who lost weight with women who gained weight.

## Methods

### Study Design & Patient Recruitment

The study was a prospective randomized controlled trial comparing a lifestyle intervention (LI) consisting of exercise and nutritional counseling with cognitive-behavior modification to usual care (UC) in overweight and obese endometrial cancer survivors. An invitation letter was sent to women included in the cancer registry from the Ireland Cancer Center at University Hospitals Case Medical Center diagnosed with stage I-II endometrial cancer from 2001–2004. The 6 month intervention was delivered to the LI group while the UC group received only an informational brochure outlining the benefits of proper nutrition and physical activity. Institutional review board approval was granted and informed consent and authorization to use protected health information (HIPAA) was obtained from all patients prior to study procedures. Feasibility, eligibility criteria, and details of the intervention have been published elsewhere [11]. Randomization assignment was stratified by BMI (<40 versus ≥ 40). Prior analysis revealed that demographics and clinical characteristics were equivalent between groups [11].

### Intervention Program

The counseling protocol was administered by the study registered dietitian (RD), primary investigator (PI), and psychologist. It followed a stepwise, phased approach using strategies outlined by social cognitive theory, indicating that the optimal intervention for a major behavior change should focus on establishing short-term goals, and enabling the person to build self-efficacy [12-16]. The intervention covered topics related to nutrition and exercise and met weekly for six weeks, biweekly for one month, and monthly for 3 months. Participants were contacted by the RD either by phone, email, or newsletter every week that the group did not meet. The psychologist met with the group during 2 of the 11 sessions and topics included cognitive and behavioral self-management strategies for weight loss and stress management. The PI met with both LI and UC participants at 3, 6 and 12 months.

### Measures

Patient demographic and clinical data was obtained at baseline and prior to randomization. QOL and self-efficacy were assessed at baseline and at 3, 6, and 12 months. Eating behavior and depression was assessed only at baseline and 12 months. QOL was measured by the ***Functional Assessment of Cancer Therapy-General ***(FACT-G), a valid and reliable questionnaire evaluating physical, functional, family-social, and emotional well-being domains [17,18]. A fatigue subscale (-F) and an endometrial symptom subscale (-En) were also used [19,20]. Functional status was measured with the ***SF-36***, a comprehensive short-form survey designed to measure functional health and well-being [21].

Depression was measured by the Beck Depression Inventory (BDI) which is well-validated and frequencly used in lifestyle research studies [22]. Self-efficacy, an individual's judgement regarding his/her ability to perform certain behaviors, was measured with the ***Weight Efficacy Life-Style (WEL) ***questionnaire. This scale specifically evaluates self-efficacy judgments specific to eating behaviors in five situational factors: negative emotions, food availability, social pressure, physical discomfort, and positive activities [23,24]. This measure has evidenced adequate psychometric properties, including internal consistency coefficients ranging from 0.76 to 0.90 [23].

Eating behavior was measured by the ***Three-Factor Eating Questionnaire (TFEQ)***, a self-assessment questionnaire developed to measure cognitive and behavioral components of eating [25,26]. Responses are aggregated into three scales, cognitive restraint, disinhibition and hunger. Cognitive restraint is designed to measure dietary restraint, that is, control over food intake in order to influence body weight and body shape. Disinhibition measures episodes of loss of control over eating, while the hunger scale is concerned with subjective feelings of hunger and food cravings. Higher numbers reflect increased restraint, decreased tendency to overeat in the presence of disinhibitors (i.e. stress, mood, alcohol) and decreased perception of hunger. All three subscales have demonstrated high internal consistency and reliability [27,28].

***Patient anthropometric data ***including weight, waist circumference, and BMI were measured at all time points manually by the study dietitian. BMI was computed as patient weight in kilograms divided by the square of their height in meters. Participants were categorized according to World Health Organization guidelines: < 18.5 (underweight), 18.5 to 24.9 (healthy weight), 25.0 to 29.9 (overweight), Class I – 30.0 to 34.9, Class II – 35.0–39.9, and Class III or morbid obesity ≥ 40.0 kg/m^2 ^[29].

### Statistical Analyses

Patient demographic, clinical variables and baseline values were compared between groups and by BMI (25.0 – 39.9 versus ≥ 40) by use of independent samples t-test or chi-square test for proportions. Primary analysis used repeated measures analysis of variance (ANOVA) with the 3, 6 and 12 month data as outcomes and the appropriate baseline measurement as a covariate to test for the main effect of group (LI versus UC, intention-to-treat analyses) on QOL outcome measures (FACT-G; physical, functional, social, emotional well-being, fatigue and endometrial subscales) and self-efficacy (WEL; negative emotions, food availability, social pressure, positive activities, physical discomfort). Change (from baseline to 12 months) in eating behavior (TFEQ) and depression measures were compared using paired samples t-test for each group and independent samples t-test for the difference in change between groups. Missing data for participants who did not complete all study assessments was handled according to the last and next method or previous row mean method as recommended by Engels et al [30]. Imputation was done on between 15–19% of values for the various QOL and eating behavior measures. The percentage of missing data for these measures was less than that imputed for weight values as some patients opted to mail these surveys back as data were self-report. Data were also analyzed using last observation carried forward and completers only approaches. There were no substantive differences among the three approaches and thus we present results of the first approach only.

QOL outcomes were also examined according to whether patients lost weight or their weight remained stable/gained over the course of the year as an ancillary stratified analyses. We were interested in examining if QOL outcomes differed between these two groups as there were some women in the UC group who lost weight and likewise women in the LI group who did not lose weight [31]. SPSS for Windows (version 14.0) was used for statistical analyses (SPSS Inc., Chicago, IL).

Approximately 25 patients per group were needed to provide 80% power to detect a difference between groups in mean weight change from baseline to twelve months of 5 kg (11 pounds) or greater, representing approximately 5% weight loss for an obese female (alpha = 0.05, two-sided, SD = 5.0) [32]. Five percent weight change is considered clinically relevant and a recommended goal for weight loss over 6 months [33,34]. Weight change was the primary endpoint of this feasibility trial and the study was powered based on this endpoint. In addition, 25 patients/group were needed to detect a large standardized effect size (d = 0.80) with power of 0.80 and a two-tailed alpha of 0.05. A large standardized effect size on the FACT-G is approximately 10 points based on a standard deviation of 12 and exceeds the 7 point MID identified for this scale [18,35].

## Results

Forty-five patients were enrolled; 23 were randomized to the LI group and 22 to the UC group. In summary, most patients were Caucasian with an average age of 55 years. As reported previously, at 12 months, the LI group lost 3.5 kg compared to a 1.4 kg gain in the UC group (p = .02) and increased their exercise by 16.4 metabolic equivalents (METS) compared to a decrease of 1.3 METS in the UC group (*p *< .001) [11]. Average time since diagnosis was 2 years and mean BMI was 42.3 kg/m^2 ^[11]. Fifty-three percent (24/45) of participants reported being very overweight during the previous ten years and 15/45 (33%) reported being moderately overweight. Patients with a BMI > 40 had increased abdominal obesity with a mean (SD) waist circumference of 125.6 (15.9) cm as compared to those with a BMI < 40 (mean 98.5 cm (8.2), p < 0.01). Waist/hip ratio did not differ between morbidly obese and BMI < 40 patients (mean 0.84 (SD = .07). Co-morbidities related to obesity (hypertension, diabetes, CVD, arthritis and metabolic syndrome) were common in these patients [11]. Five patients had prior bariatric surgery prior to enrollment (LI; 4, UC: 1) with surgeries performed 3–6 years before the study began.

### Baseline differences based on body mass index

Baseline QOL (FACT-G, SF-36), BDI, WEL and TFEQ scores did not differ between the LI and UC groups. However significant differences were observed when patients were categorized according to BMI (Table 1). Total QOL (FACT-G) was not significantly lower in morbidly obese women [BMI ≥ 40: 78.0 (SD = 14.4) vs. BMI < 40: 83.7 (SD = 11.4); *p *= 0.14], however the difference was consistent with minimally important differences (5–6 points) [18]. SF-36 physical composite score was decreased in morbidly obese women (p = 0.04). At baseline 7/45 (15%) patients reported mild depressive symptoms, and 4/45 (9%) reported moderate depressive symptomatology. Beck depression score did not differ according to BMI (Table 1).

**Table 1:** Quality of life, depression, self-efficacy and eating inventory scores at baseline (n = 45)

**Variables**	**Overall** **(n = 45)**	**Body mass** **index < 40** **(n = 23)**	**Body mass** **index ≥ 40** **(n = 22)**	p value *
**Quality of Life**				
FACT-G (0–108)	80.1 (12.1)	83.7 (9.6)	78.0 (14.1)	0.14

Physical well-being (0–28)	23.9 (3.2)	24.8 (3.2)	23.2 (3.3)	0.12

Social well-being (0–24)	16.5 (3.1)	16.6 (2.6)	16.5 (3.2)	0.90

Emotional well-being (0–24)	18.6 (4.1)	19.4 (3.9)	17.9 (4.4)	0.27

Functional well-being (0–28)	21.2 (5.4)	22.9 (4.0)	20.4 (5.4)	0.10

Endometrial (0–64)	53.5 (5.8)	52.8 (5.0)	54.6 (6.6)	0.32

Fatigue (0–52)	37.9 (9.7)	40.2 (9.7)	36.2 (9.8)	0.19

Physical composite score, SF36	43.9 (10.7)	47.1 (8.7)	40.6 (11.7)	**0.04**

Mental composite score, SF36	50.0 (10.9)	50.6 (9.8)	49.3 (12.2)	0.70

**Beck depression inventory (0–63)**	10.3 (7.9)	8.8 (6.5)	11.4 (9.0)	0.29

**Weight self-efficacy**				
Negative Emotions (0–36)	18.8 (10.8)	20.4 (9.7)	15.5 (10.4)	0.12
Food Availability (0–36)	18.3 (9.3)	20.0 (8.8)	15.4 (8.9)	0.09
Social Pressure (0–36)	24.2 (8.7)	25.0 (7.2)	22.3 (9.7)	0.31
Physical Discomfort (0–36)	26.5 (8.3)	28.8 (5.8)	23.4 (9.4)	**0.03**
Positive Activities (0–36)	24.3 (7.3)	25.5 (5.0)	22.3 (8.5)	0.15
Total (0–180)	112.1 (36.8)	119.6 (26.7)	98.8 (39.0)	**0.05**

**Three-factor eating questionnaire**				
Restraint (0–21)	10.3 (4.9)	12.8 (4.4)	7.5 (3.8)	**< 0.01**
Disinhibition (0–16)	7.9 (3.6)	7.3 (3.8)	9.2 (3.0)	0.10
Perceived Hunger (0–14)	5.6 (3.4)	5.6 (3.4)	6.3 (3.6)	0.50

* Independent samples Student's t-test. Data are presented as the mean (standard deviation) FACT-G, Functional Assessment of Cancer Therapy-General; SF-36, Short-form Medical Outcomes Survey

In regards to eating patterns, morbidly obese patients had significantly lower self-efficacy when feeling physical discomfort and decreased total self-efficacy (WEL) score. Restraint on the TFEQ questionnaire was decreased in patients with BMI > 40 as compared to BMI < 40.

### Effects of the lifestyle intervention (Intention-to-treat analysis)

Repeated measures ANOVA with baseline measurements as a covariate and 3, 6 and 12 months as outcomes revealed no group (LI versus UC) effects for QOL outcomes (Table 2). There was a significant group effect for self-efficacy related to social pressure and restraint improved in the LI group. The UC group had a significant change in depression from baseline. Change scores for depression or TFEQ, compared by independent samples t-test, however did not differ by group.

**Table 2:** Effects of the lifestyle intervention on QOL and clinical outcomes (intention-to-treat analysis)

**Variables**	**Baseline**	**3 mos**	**6 mos**	**12 mos**	Group effect P*
**Primary Quality of life outcome**					
FACT-G (0–108)					0.37
Lifestyle intervention	80.6 (12.7)	81.1 (14.0)	82.4 (14.5)	80.1 (14.2)	
Usual care	79.5 (13.1)	80.1 (15.5)	81.8 (13.2)	82.2 (14.9)	

**Secondary QOL outcomes**					
Physical well-being (0–28)					0.84
LI	23.9 (3.4)	23.6 (4.3)	24.0 (4.4)	23.3 (4.3)	
UC	23.9 (3.0)	23.3 (4.5)	24.2 (4.1)	23.7 (4.1)	

Functional well-being (0–28)					
LI	21.3 (5.2)	22.0 (5.6)	22.0 (5.7)	21.2 (6.2)	0.44
UC	21.0 (5.7)	22.0 95.6)	22.0 (5.0)	22.0 (6.1)	

Emotional well-being (0–24)					
LI	18.3 (4.4)	19.1 (4.5)	19.3 (4.5)	18.8 (4.5)	0.98
UC	18.9 (3.9)	19.2 (4.0)	19.5 (4.1)	20.0 (3.7)	

Social/family well-being (0–28)					
LI	17.2 (2.7)	16.6 (3.0)	17.2 (2.7)	16.9 (2.6)	0.17
UC	15.7 (3.3)	15.6 (3.4)	16.3 (3.0)	16.6 (3.4)	

Fatigue (0–52)					
LI	37.5 (10.3)	38.5 (11.3)	39.2 (11.6)	38.5 (11.1)	0.99
UC	38.2 (9.2)	39.0 (10.4)	39.2 (11.5)	40.0 (9.3)	

Endometrial subscale (0–64)					
LI	54.4 (5.4)	54.9 (6.7)	55.5 (6.8)	55.0 (6.7)	0.46
UC	52.3 (6.2)	51.9 (6.9)	52.6 (6.9)	53.6 (6.2)	

**Weight self-efficacy**					
Negative emotion (0–36)					0.87
LI	17.0 (10.4)	21.7 (9.8)	23.1 (9.7)	21.9 (9.1)	
UC	19.9 (10.8)	24.9 (9.9)	22.8 (10.0)	23.9 (10.9)	
					
Food Availability (0–36)					0.49
LI	17.5 (8.5)	23.1 (8.9)	23.2 (9.9)	22.4 (10.1)	
UC	18.8 (10.3)	23.6 (9.8)	21.0 (9.5)	23.2 (9.9)	
					
Social pressure (0–36)					**0.03**
LI	23.9 (8.1)	28.2 (7.0)	28.4 (7.5)	27.9 (7.9)	
UC	23.9 (9.3)	26.1 (8.5)	23.3 (8.0)	26.0 (7.3)	
					
Positive activities (0–36)					
LI	23.2 (5.7)	27.5 (4.8)	27.8 (4.2)	27.3 (4.6)	0.10
UC	25.1 (8.5)	27.5 (8.1)	24.9 (7.7)	27.9 (5.0)	
					
Physical discomfort (0–36)					0.38
LI	25.8 (8.4)	28.5 (7.1)	29.1 (6.6)	29.7 (6.7)	
UC	26.8 (8.2)	29.1 (6.5)	27.6 (6.7)	29.0 (6.4)	
					
Total (0–180)					0.19
LI	107.4 (31.5)	129.1 (30.4)	131.5 (31.7)	129.3 (31.3)	
UC	114.5 (40.9)	131.1 (39.4)	119.6 (37.7)	130.0 (35.6)	

**Beck depression inventory**					
LI	9.7 (6.8)	-	-	8.6 (7.2)	0.16
UC	10.6 (8.9)	-	-	8.3 (7.4)	**0.02**

**Three-factor eating questionnaire**					
Restraint (0–21)		-	-		
LI	10.0 (4.8)	-	-	11.9 (5.1)	**0.02**
UC	10.3 (5.2)			11.1 (4.9)	0.24
					
Disinhibition (0–16)		-	-		
LI	8.8 (2.8)	-	-	8.4 (3.3)	0.44
UC	7.1 (4.1)			7.0 (4.1)	0.68
					
Hunger (0–14)		-	-		
LI	6.2 (3.3)			6.0 (2.7)	0.67
UC	5.1 (3.6)			4.2 (2.6)	0.13

Data are presented as the mean (standard deviation). LI (n = 23), UC (n = 22)

* p value for repeated measures ANOVA with baseline measurement as covariate and 3, 6 and 12 months as outcomes or paired samples t-test (eating inventory and Beck depression inventory)

Abbreviations: QoL, quality of life; FACT-G, Functional Assessment of Cancer Therapy-General; SF-36, Short-form Medical Outcomes Survey; BDI, Beck Depression Inventory; WEL, weight efficacy lifestyle; TFEQ, three-factor eating questionnaire; BMI, body mass index

### Association between weight loss and outcomes (exploratory analysis)

Table 3 presents an ancillary analysis for QOL, self-efficacy, depression and eating behavior outcomes. For this analysis we compared women who lost weight (WL; n = 21) versus those whose weight was the same or who gained weight (WG; n = 24) from baseline to twelve months. There was a significant effect (WL versus WG) for emotional well-being QOL, self-efficacy related to negative emotions, food availability, and physical discomfort as the WL group had higher scores. For self-efficacy related to negative emotions, there was a mean increase of 8.9 in women who lost weight versus 0.6 in those whose weight was stable/gained. Similarly, for self-efficacy related to food availability and physical discomfort, the weight loss group had an increase of 7.9 and 5.3 versus 1.9 and 1.0 in women who did not lose weight. Women who lost weight had improvement in depression (2.3 versus 1.2) and TFEQ restraint score (2.8 versus 0.1). Women who lost weight also had a lower disinhibition score at 12 months. Group differences for change in TFEQ restraint score were observed using independent samples t-test for comparison of change scores between WL and WG (p = .01), but not for depression.

**Table 3:** Effects of weight loss on QOL and clinical outcomes (ancillary analyses)

**Variables**	**Baseline**	**3 mos**	**6 mos**	**12 mos**	Group effect P*
**Primary QoL outcome**					
FACT-G					0.26
WL	81.4 (14.0)	83.5 (15.7)	84.8 (14.7)	82.3 (15.4)	
WG	78.9 (11.7)	78.1 (13.3)	79.8 (12.6)	80.1 (13.8)	

**Secondary QoL outcomes**					
Physical well-being (0–28)					0.79
WL	24.7 (3.3)	24.5 (4.6)	24.7 (4.7)	24.2 (4.8)	
WG	23.1 (3.0)	22.5 (4.0)	23.5 (3.7)	22.8 (3.5)	

Functional well-being (0–28)					
WL	21.9 (5.6)	22.8 (5.3)	23.3 (5.0)	21.9 (6.1)	0.65
WG	20.5 (5.2)	21.2 (5.8)	20.8 (5.4)	21.2 (6.2)	

Emotional well-being (0–24)					
WL	18.2 (4.3)	20.0 (4.1)	20.0 (4.3)	19.5 (4.2)	**0.02**
WG	18.9 (4.0)	18.4 (4.2)	18.9 (4.2)	19.3 (4.1)	

Social/family well-being (0–28)					
WL	16.6 (3.2)	16.2 (3.4)	17.0 (2.8)	16.7 (2.8)	0.98
WG	16.4 (3.0)	16.0 (3.0)	16.5 (2.9)	16.8 (3.2)	

Fatigue (0–52)					
WL	39.0 (10.2)	41.6 (10.5)	41.4 (12.1)	42.0 (10.5)	0.07
WG	36.9 (9.3)	36.3 (10.5)	37.3 (10.7)	36.9 (9.5)	

Endometrial subscale (0–64)					
WL	54.3 (6.6)	54.3 (7.7)	55.2 (8.1)	55.5 (6.9)	0.55
WG	52.8 (5.1)	52.7 (6.2)	53.1 (5.7)	53.3 (5.9)	

**WEL**					
Negative emotions (0–36)					**< 0.01**
WL	16.7 (10.9)	24.3 (10.5)	25.6 (9.3)	25.6 (9.7)	
WG	20.0 (10.3)	22.4 (9.5)	20.5 (3.3)	20.6 (9.8)	
					
Food Availability (0–36)					**0.03**
WL	16.5 (10.4)	24.0 (9.6)	23.8 (9.9)	24.4 (10.4)	
WG	19.5 (8.3)	22.8 (9.0)	20.7 (9.4)	21.4 (9.4)	
					
Social pressure (0–36)					0.20
WL	25.2 (9.2)	28.4 (7.5)	28.1 (7.5)	29.1 (7.2)	
WG	22.7 (8.1)	26.1 (8.0)	23.9 (8.2)	25.2 (7.6)	
					
Positive activities (0–36)					0.10
WL	23.0 (7.9)	27.5 (6.2)	27.5 (5.3)	27.8 (5.0)	
WG	25.1 (6.5)	27.5 (6.9)	25.4 (7.0)	27.4 (4.6)	
					
Physical discomfort (0–36)					
WL	25.5 (9.2)	29.6 (7.0)	30.0 (6.9)	30.8 (7.1)	**0.01**
WG	27.0 (7.4)	28.1 (6.5)	26.9 (6.2)	28.0 (5.7)	
					
Total (0–180)					**<0.01**
WL	106.9 (37.4)	133.8 (36.0)	135.0 (33.8)	137.6 (33.9)	
WG	114.3 (35.5)	126.9 (33.9)	117.4 (34.4)	122.6 (31.4)	

**Beck depression inventory**					
WL	9.3 (7.0)	-	-	7.0 (6.5)	**0.03**
WG	10.9 (8.8)	-	-	9.7 (7.9)	0.14

**TFEQ**					
Restraint (0–21)					
WL	10.1 (4.5)	-	-	12.9 (4.2)	**<0.01**
WG	10.2 (5.4)	-	-	10.3 (5.3)	0.74
					
Disinhibition (0–16)					
WL	7.8 (3.0)	-	-	6.9 (3.3)	**0.05**
WG	8.1 (4.1)	-	-	8.4 (4.1)	0.48
					
Hunger (0–14)					
WL	6.1 (3.8)	-	-	5.1 (2.5)	0.21
WG	5.3 (3.1)	-	-	5.1 (3.1)	0.51

Data are presented as the mean (standard deviation).

WL, n = 21; WG, n = 24

* p value for repeated measures ANOVA with baseline measurement as covariate and 3, 6 and 12 months as outcomes or paired samples t-test (eating inventory and Beck depression inventory)

Abbreviations: QoL, quality of life; FACT-G, Functional Assessment of Cancer Therapy-General; SF-36, Short-form Medical Outcomes Survey; BDI, Beck Depression Inventory; WEL, weight efficacy lifestyle; TFEQ, three-factor eating questionnaire; BMI, body mass index

## Discussion

Lifestyle interventions are necessary for survivorship in obese endometrial cancer patients as they are at risk for premature death not due to cancer but secondary to poor cardiovascular health. The majority of patients in this study were morbidly obese and had lower physical health-related scores. The lifestyle intervention did not have any effect on global QOL outcomes, however self-efficacy, emotional well-being and certain eating behaviors improved with weight loss. Although the intervention was efficacious in promoting weight loss, the lack of influence on QOL was contrary to the hypothesis. It may be that a longer intervention, greater weight loss or increased exercise is needed to improve QOL. Functional, and psychological-related changes may require a longer term investment in a healthy lifestyle before reaching significance.

Social cognitive theory [36] is a well-established explanation of health behavior change, and is frequently utilized in dietary and physical activity lifestyle interventions [37-39]. Social cognitive theory explains health behaviors in terms of reciprocal relationships between behavior, personal factors and environmental influences. The power of circumstance, of being diagnosed with endometrial cancer, can potentially launch new life courses, change a person's perception of their environment, improve health behavior-related expectations and influence reciprocal determinism. Thus, endometrial cancer patients may be amenable to a "teachable moment."

Self-efficacy is the social cognitive theory concept that represents one's judgment about her ability to successfully engage in a particular behavior and overcome barriers to achieve change [36]. Toobert et al performed a lifestyle intervention trial in women with CVD and found that fat intake decreased as their self-efficacy scores increased [40]. This research and those of our collaborators supports self-efficacy as a theoretical model and a possible mediator to improve lifestyle change in the obese [41,42]. We found increased self-efficacy as related to negative emotions, food availibiltiy and physical discomfort in those women who lost weight during the year. In addition, self-efficacy scores at twelve months remained increased, six months after the intervention had concluded. In terms of eating behavior, restraint was also improved in patients who lost weight. Weight losers, however had a lower disinhibition score, indicating an increase likelihood to overeat in the presence of disinhibitors. This was an unexpected finding and may indicate that there are still certain triggers that are evident and more attention to these is possibly needed. Different populations including endometrial cancer survivors, may need more intense interventions in order to change morbid patterns [24].

Limitations of this study include its small sample size, and lack of racial heterogeneity. There was a greater number of patients in the intervention group who had a prior history of bariatric surgery. These patients may have better self-efficacy and eating behavior patterns that could influence results though the number of patients is too small to make any conclusions. However, the findings should trigger clinicians to focus on positive lifestyle change, improving self-efficacy and decreasing co-morbidity in endometrial cancer survivors. An additional limitation was potential contamination of the control group, as depression improved in the control group over time. This may have been influenced by all study patients participating in an orientation meeting, in addition to meeting with the principal investigator at the 3 measurement time points. It may be that simple increased physician contact (without teaching) can improve outcome measures. Others have questioned whether improving QOL could be attributable to the increased attention (or increasing self-efficacy) given to cancer patients involved in exercise interventions [43]. QOL may improve temporally as patients experience more years of survivorship and travel farther from the challenging time of diagnosis. In addition, we can also hypothesize that the weight loss observed was not large enough to see a change in QOL. However, given that this was a feasibility study the results are helpful in designing a future intervention trial.

## Conclusion

This pilot lifestyle intervention had no effect on quality of life, or depression but did improve self-efficacy and restraint. A substantial amount of endometrial cancer survivors are surviving their cancer, however, they are succumbing to other diseases correlated with obesity. The Gynecologic Oncology Group is considering adding QOL measures to their next large prospective endometrial cancer trial with long term follow up. Future directions will also consist of the measurement of 5 year outcomes in this study population [44]. Goals of upcoming projects are to decrease co-morbidity and increase overall survival in endometrial cancer survivors.

## Competing interests

The authors declare that they have no competing interests.

## Authors' contributions

VVG, HG, MBK, JJ, EL and KC conceived of the study, and participated in its design and coordination. VVG, HG, and MBK implemented the study and were responsible for day to day conduct of the study. VVG, HG, and KC analyzed the data. VVG, HG, and KC drafted the manuscript; MBK, JJ and EL provided critical review. All authors read and approved the final manuscript.

## References

[B1] Jemal A, Siegel R, Ward E, Murray T, Xu J, Thun MJ (2007). Cancer Statistics, 2007. CA Cancer J Clin.

[B2] von Gruenigen VE, Gil KM, Frasure HE, Grandon M, Hopkins MP, Jenison EL (2005). Complementary medicine use, diet and exercise in endometrial cancer survivors. J Cancer Integ Med.

[B3] Anderson B, Connor JP, Andrews JI, Davis CS, Butler RE, Sorosky JL (1996). Obesity and prognosis in endometrial cancer. Am J Obstet Gynecol.

[B4] Kennedy AW, Austin JM, Look KY, Munger CB (2000). The Society of Gynecologic Oncologists Outcomes Task Force. Study of endometrial cancer: Initial experiences. Gynecol Oncol.

[B5] Everett E, Tamini H, Geer B, Swisher E, Paley P, Mandel L (2003). The effect of body mass index on clinical/pathologic features, surgical morbidity, and outcome in patients with endometrial cancer. Gynecol Oncol.

[B6] Mokdad AH, Ford ES, Bowman BA, Dietz WH, Vinicor F, Bales VS (2003). Prevalence of obesity, diabetes, and obesity-related health risk factors, 2001. JAMA.

[B7] Calle EE, Rodriguez C, Walker-Thurmond K, Thun MJ (2003). Overweight, obesity, and mortality from cancer in a prospectively studied cohort of U.S. adults. N Engl J Med.

[B8] von Gruenigen VE, Tian C, Frasure HE, Waggoner SE, Barakat RR (2006). Treatment effects, disease recurrence and survival as related to obesity in women with early endometrial carcinoma: A Gynecologic oncology group study. Cancer.

[B9] von Gruenigen VE, Frasure HE, Grandon M, Jenison EL, Hopkins MP (2005). Impact of Obesity and Age on Quality of Life in Gynecologic Surgery. Am J Obstet Gynecol.

[B10] Courneya KS, Karvinen KH, Campbell KH, Pearcey RG, Dundas G, Capstick V (2005). Associations among exercise, body weight, and quality of life in a population-based sample of endometrial cancer survivors. Gynecol Oncol.

[B11] von Gruenigen VE, Courneya KS, Gibbons HE, Waggoner SE, Kavanagh MB, Lerner E (2008). Feasibility and effectiveness of a lifestyle intervention program in obese endometrial cancer patients. Gynecol Oncol.

[B12] Pierce JP, Faerber S, Wright FA, Rock CL, Newman V, Flatt SW, Kealey S (2002). A randomized trial of the effect of a plant-based dietary pattern on additional breast cancer events and survival: the women's healthy eating and living (WHEL) study. Control Clin Trials.

[B13] Holmes MD, Chen WY, Feskanich D, Kroenke CH, Colditz GA (2005). Physical activity and survival after breast cancer diagnosis. JAMA.

[B14] Chlebowski RT, Nixon DW, Blackburn GL, Jochimsen P, Scanlon EF, Insull W (1987). A breast cancer Nutrition Adjuvant Study (NAS): Protocol design and initial patient adherence. Breast Cancer Res Treat.

[B15] Chlebowski RT, Blackburn GL, Thomson CA, Nixon DW, Shapiro A, Hoy MK (2006). Dietary fat reduction and breast cancer outcome: interim efficacy results from the Women's Intervention Nutrition Study. J Natl Cancer Inst.

[B16] Demark-Wahnefried W, Clipp EC, Morey MC, Pieper CF, Sloane R, Snyder DC (2006). Lifestyle intervention development study to improve physical function in older adults with cancer: Outcomes from Project LEAD. J Clin Oncol.

[B17] Cella DR, Tulsky DS, Gray G, Sarafin B, Linn E, Bonomi A (1993). The Functional Assessment of Cancer Therapy scale: development and validation of the general measure. J Clin Oncol.

[B18] Webster K, Cella D, Yost K (2003). The Functional Assessment of Chronic Illness Therapy (FACIT) Measurement System: properties, applications, and interpretation. Health Qual Life Outcomes.

[B19] Yellen SB, Cella DF, Webster K, Blendowski C, Kaplan E (1997). Measuring fatigue and other anemia-related symptoms with the Functional Assessment of Cancer Therapy (FACT) measurement system. J Pain Symptom Manage.

[B20] FACIT Functional Assessment of Chronic Illness Therapy. View questionnaires.

[B21] Ware JE, Sherbourne CD (1992). The MOS 36-item short-form health survey (SF-36). I. Conceptual framework and item selection. Med Care.

[B22] Beck AT, Steer RA, Brown GK (1996). Beck Depression Inventory.

[B23] Clark MM, Abrams DB, Niaura RS (1991). Self-efficacy in weight management. J Consult Clin Psych.

[B24] Clark MM, Forsyth LH, Lloyd-Richardson EE, King TK (2000). Eating self-efficacy and binge eating disorder in obese women. J Appl Biobehav Res.

[B25] Stunkard AJ, Messick S (1985). The three-factor eating questionnaire to measure dietary restraint, disinhibition, and hunger. J Psycholsom Res.

[B26] Foster GD, Wadden TA, Swain RM, Stunkard AJ, Platte P, Vogt RA (1998). The eating inventory in obese women: clinical correlates and relationship to weight loss. Int J Obes Relat Metab Disord.

[B27] Allison DB, Kalinsky LB, Gorman BS (1992). The comparative psychometric properties of three measures of dietary restraint. Psych Assess.

[B28] Laessle RG, Tuschl RJ, Kotthaus BC, Pirke KM (1989). A comparison of the validity of three scales for the assessment of dietary restraint. J Abnorm Psychol.

[B29] Clinical guidelines on the identification, evaluation, and treatment of overweight and obesity in adults: the evidence report. National Heart Lung and Blood Institute.

[B30] Engels JM, Diehr P (2003). Imputation of missing longitudinal data: a comparison of methods. J Clin Epidemiol.

[B31] Courneya KS, Friedenreich CM, Quinney HA, Fields ALA, Jones LW, Fairey AS (2003). A randomized trial of exercise and quality of life in colorectal cancer survivors. European Journal of Cancer Care.

[B32] Andersen RE, Wadden TA, Bartlett SJ, Zemel B, Verde TJ, Franckowial SC (1999). Effects of lifestyle activity vs structured aerobic exercise in obese women: A randomized trial. JAMA.

[B33] McTigue KM, Harris R, Hemphill B, Lux L, Sutton S, Bunton AJ (2003). Screening and interventions for obesity in adults: Summary of the evidence for the U.S. preventive services task force. Ann Intern Med.

[B34] Jakicic JM, Wing RR, Winters-Hart C (2002). Relationship of physical activity to eating behaviors and weight loss in women. Med Sci Sports Exerc.

[B35] Cella D, Hahn EA, Dineen K (2002). Meaningful change in cancer-specific quality of life scores: differences between improvement and worsening. Qual Life Res.

[B36] Bandura A (1977). Self-efficacy: Toward a unifying theory of behavioral change. Psych Rev.

[B37] Luepker RV, Perry CL, McKinlay SM, Nader PR, Parcel GS, Stone EJ (1996). Outcomes of a field trial to improve children's dietary patterns and physical activity. The Child and Adolescent Trial for Cardiovascular Health. CATCH collaborative group. JAMA.

[B38] Rogers LQ, Shah P, Dunnington G, Grieve A, Shanmugham A, Dawson B, Courneya KS (2005). Social cognitive theory and physical activity during breast cancer treatment. Oncology Nursing Forum.

[B39] Rogers LQ, Matevey C, Hopkins-Price P, Shah P, Dunnington G, Courneya KS (2004). Exploring social cognitive theory constructs for promoting exercise among breast cancer patients. Cancer Nursing.

[B40] Toobert DJ, Glasgow RE, Nettekoven LA, Brown JE (1998). Behavioral and psychosocial effects of intensive lifestyle management for women with coronary heart disease. Patient Education and Counseling.

[B41] Vallance JKH, Courneya KS, Plotnikoff RC, Yasui Y, Mackey JR (2007). Effects of print materials and step pedometers on physical activity and quality of life in breast cancer survivors: A randomized controlled trial. J Clin Oncol.

[B42] Karvinen KH, Courneya KS, Campbell KL, Pearcey RG, Dundas G, Capstick V (2007). Correlates of exercise motivation and behavior in a population-based sample of endometrial cancer survivors: an application of the Theory of Planned Behavior. Int J Behav Nutr Phys Act.

[B43] Daley AJ, Crank H, Sexton JM, Mutrie N, Coleman R, Roalfe A (2007). Randomized trial of exercise therapy in women treated for breast cancer. J Clin Oncol.

[B44] Demark-Wahnefried W, Aziz NM, Rowland JH, Pinto BM (2005). Riding the crest of the teachable moment: Promoting long-term health after the diagnosis of cancer. J Clin Oncol.

